# Supplementary material for: Xist-mediated silencing requires additive functions of SPEN and Polycomb together with differentiation-dependent recruitment of SmcHD1
Source: Cell Rep. 2022 Mar 17;39(7):110830. doi: 10.1016/j.celrep.2022.110830 (PMC9637994; doi:10.1016/j.celrep.2022.110830)
Supplement: Document S1. Figures S1–S7 and Table S1 [file mmc1.pdf]

Cell Reports, Volume *39*

Supplemental information

Xist-mediated silencing requires additive functions of SPEN and Polycomb together with differentiation-dependent recruitment of SmcHD1

Joseph S. Bowness, Tatyana B. Nesterova, Guifeng Wei, Lisa Rodermund, Mafalda Almeida, Heather Coker, Emma J. Carter, Artun Kadaster, and Neil Brockdorff

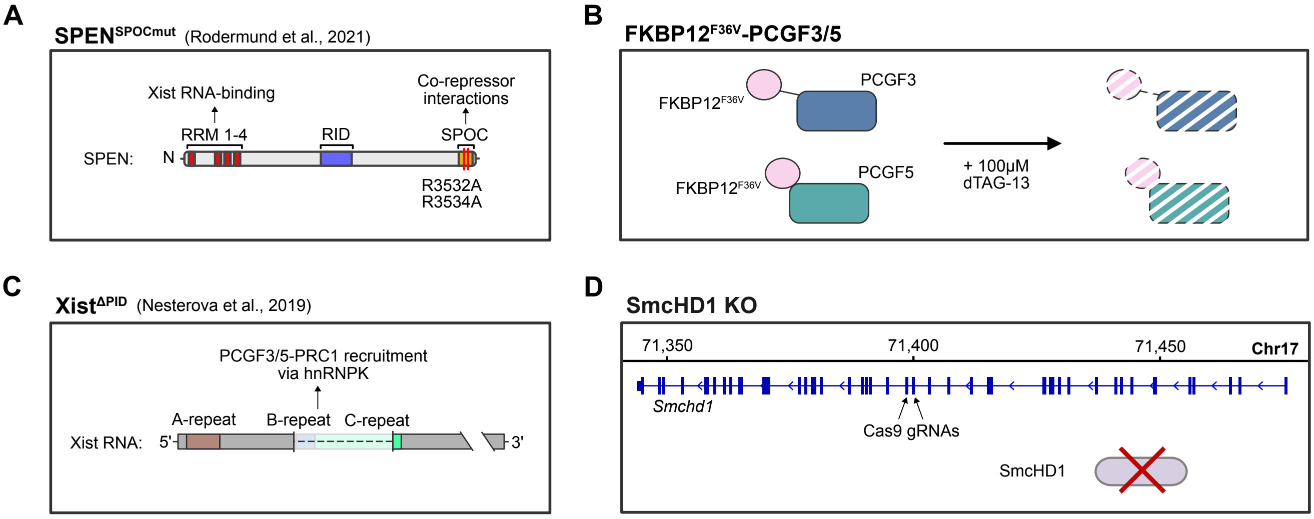

Figure S1 Schematics of mutant mESC lines discussed in this study. Related to Figures 1, 2 and 6.

(A) SPEN^{SPOCmut}: Point mutants in the SPEN SPOC domain abolish interactions with corepressors such as the SMRT/NCoR complex. SPEN protein retains its ability to bind Xist RNA via RRM (RNA Recognition Motif) domains.

(B) FKBP12^{F36V}-PCGF3/5: Sequences encoding the FKBP12^{F36V} degron tag were inserted into both endogenous *Pcgf3* and *Pcgf5* loci by CRISPR/Cas9-facilitated homologous recombination to generate homozygous N-terminal fusion proteins. The PCGF3 fusion protein contains a flexible GGSGG linker between the tag and endogenous protein which is not present for PCGF5. Upon addition of 100 μM dTAG-13 fusion proteins are rapidly degraded by the ubiquitin-proteasome system.

(C) Xist$^{\triangle PID}$: A deletion in the *Xist* locus removes the B-repeat and the majority of the C-repeat regions from Xist RNA, and thus abolishes Polycomb recruitment by Xist, which occurs via hnRNPK to PCGF3/5-PRC1 complexes.

(D) SmcHD1 KO: Knockout cell lines with no production of SmcHD1 protein were generated by CRISPR/Cas9-mediated mutagenesis of the endogenous *Smchd1* gene. More details on specific mutations is provided in Figure S7A.

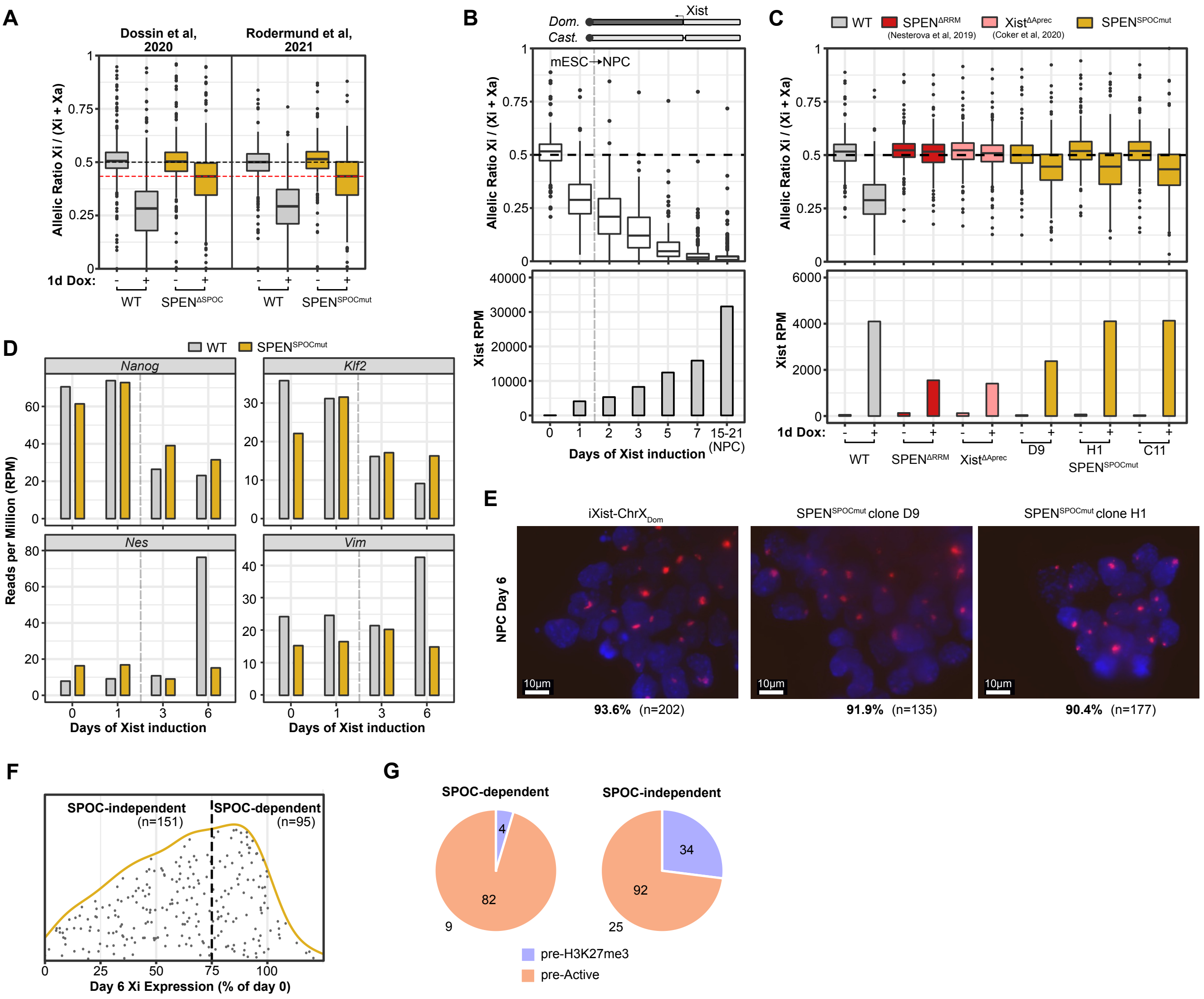

Figure S2 Characterization of iXist-ChrX$_{Dom}$ SPEN^{SPOCmut}. Related to Figure 1.

(A) Boxplots comparing allelic X-linked gene expression between SPEN^{SPOCmut} lines in our previous study (Rodermund et al., 2021) and a larger deletion of the SPEN SPOC domain introduced into a similar model cell line of XX mESCs with inducible Xist (Dossin et al., 2020).

(B) Boxplots summarizing allelic ChrRNA-seq analysis of X-linked gene expression in the iXist-ChrX$_{Dom}$ cell line. Timepoints in mESCs with and without Xist induction (day 1 and day 0 respectively) are shown alongside later timepoints of Xist induction with NPC differentiation. Data from NPC timecourse experiments are averaged (n=2). Relative levels of chromatin-associated Xist RNA for each time point are shown below.

(C) Boxplots summarizing allelic ChrRNA-seq analysis of X-linked gene expression in iXist-ChrX$_{Dom}$ WT, SPEN$^{\Delta RRMmut}$ (Nesterova et al., 2019), Xist$^{\Delta Aprec}$ (Coker et al., 2020) and three independent SPEN^{SPOCmut} clones after 1 day of Xist induction in mESCs. SPEN$^{\Delta RRMmut}$ and Xist$^{\Delta Aprec}$ boxes are averaged from two replicate clones. Relative levels of chromatin-associated Xist RNA for each time point are shown below.

(D) Relative levels of marker gene expression for samples presented in Figure 1A,B.

(E) Xist RNA-FISH in iXist-ChrX$_{Dom}$ and two derivative SPEN^{SPOCmut} clones performed after six days of Xist induction with NPC differentiation. Scoring of the percentage of cells containing Xist domains is indicated below each representative image.

(F) Density plot illustrating the methodology used to define a subset of genes that demonstrate greater silencing in SPEN^{SPOCmut} cells. Genes, represented by individual dots, were defined as 'SPOC-dependent' if the allelic ratio at day 6 is $>$ 75% of the initial allelic ratio in uninduced day 0 samples.

(G) Pie charts illustrating the proportions of SPOC-independent and SPOC-dependent genes in 'pre-Active' and 'pre-H3K27me3' gene categories, as defined in (Nesterova et al., 2019). 34 genes were not amenable to ChromHMM analysis in our previous study.

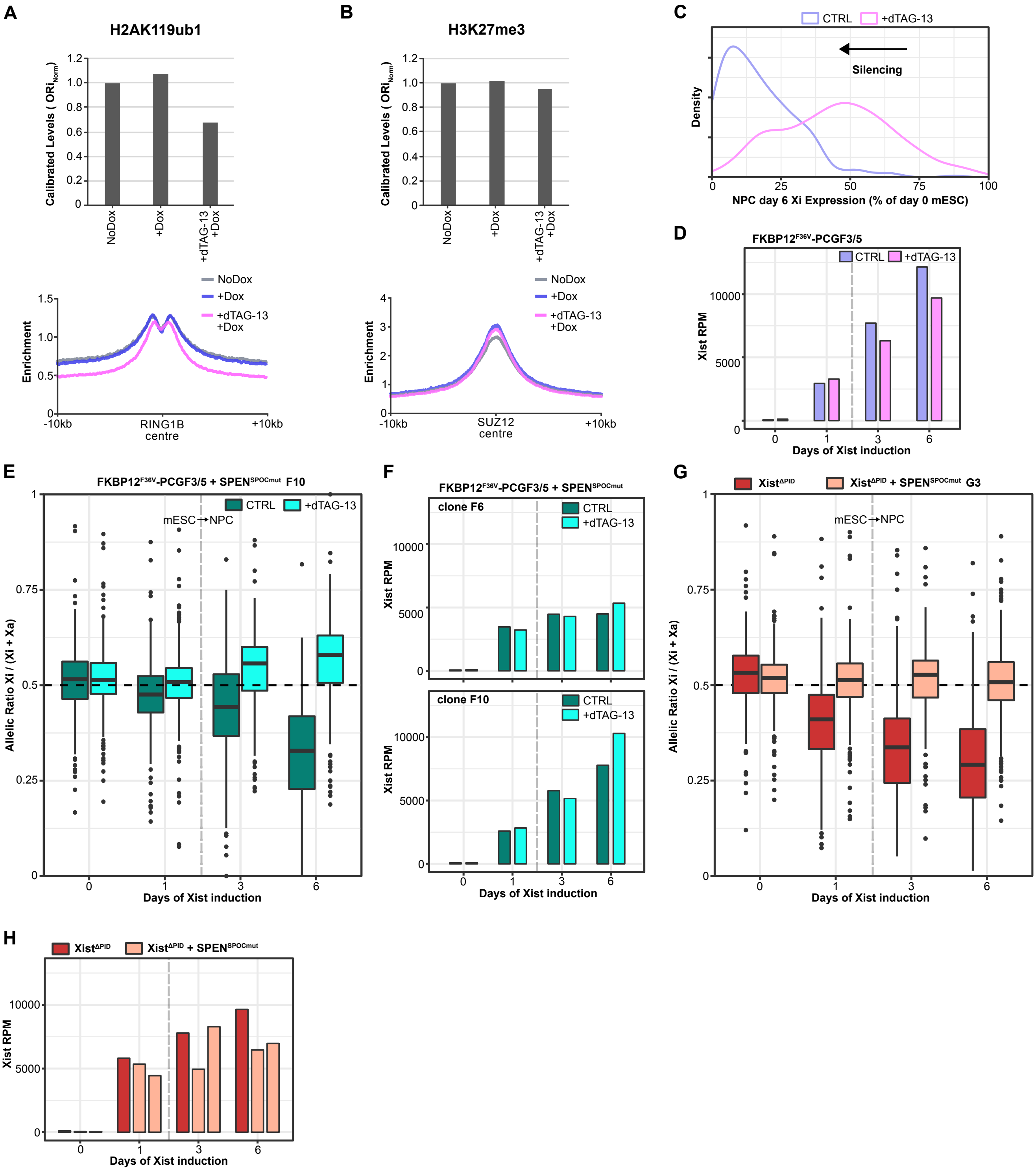

Figure S3 Characterization of iXist-ChrX$_{Dom}$ FKBP12^{F36V}-PCGF3/5 and combined Polycomb + SPEN^{SPOCmut} lines. Related to Figure 2.

(A) (above) Calibrated global levels of H2AK119ub1 from ChIP-seq of FKBP12^{F36V}-PCGF3/5 with exogenous spike-in of *Drosophila* cells, averaged from two replicate ChIP-seq experiments (see STAR Methods). (below) Meta-profiles of H2AK119ub1 enrichment centred on PRC1 target regions defined by RING1B ChIP-seq peak centres in mESCs (Fursova et al., 2019).

(B) As (A) for H3K27me3 and PRC2 regions defined by SUZ12 ChIP-seq peak centres in mESCs (Fursova et al., 2019).

(C) Density plot illustrating gene silencing after six days of differentiation in FKBP12^{F36V}-PCGF3/5 with or without dTAG-13 treatment for 12 hours prior to Xist induction.

(D) Relative levels of chromatin-associated Xist for each sample of the boxplots in Figure 2C.

(E) As Figure 2D for FKBP12^{F36V}-PCGF3/5 + SPEN^{SPOCmut} clone G3. The upward skew of allelic ratio for NPC-differentiated samples in this clone is interpreted to be a consequence of selection for cells in the population which have spontaneously eliminated the *Castaneous* X chromosome.

(F) Relative levels of chromatin-associated Xist for each FKBP12^{F36V}-PCGF3/5 + SPEN^{SPOCmut} clone, comparing untreated cells and cells treated for 12 hours with dTAG-13 prior to Xist induction cultured in parallel.

(G) As Figure 2E for Xist$^{\Delta PID}$ + SPEN^{SPOCmut} clone G, which is tetraploid and contains two copies of each ChrX allele (see Figure S4D).

(H) Relative levels of chromatin-associated Xist in ChrRNA-seq data sets from Xist$^{\Delta PID}$ and Xist$^{\Delta PID}$ + SPEN^{SPOCmut} clones presented in Figure 2E and Figure S3G.

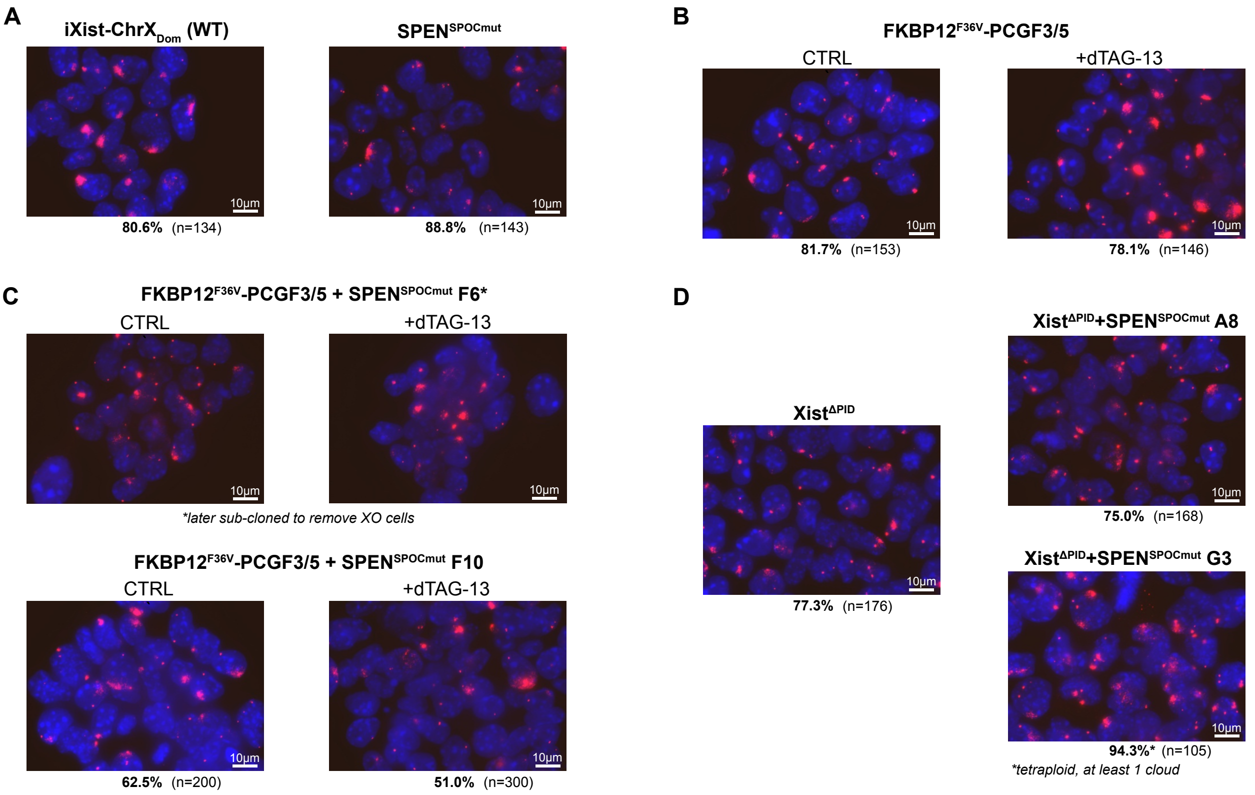

Figure S4 Efficient Xist induction and cloud formation in SPEN^{SPOCmut} and Polycomb mutant lines. Related to Figure 3.

Xist RNA-FISH images collected from mESCs after 1 day of Xist induction for mutant cell lines presented in Figures 1 and 2 of this study. For the FKBP12^{F36V}-PCGF3/5 line and clonal derivatives, dTAG-13 treatment was applied 12 hours prior to Xist induction. The percentage of cells containing Xist territories is indicated below each representative image. RNA-FISH characterization of Xist induction in FKBP12^{F36V}-PCGF3/5 + SPEN^{SPOCmut} clone F6 (C) revealed a sub-population of XO cells with only one Xist/Tsix cloud/foci. This clone was therefore sub-cloned prior to the ChrRNA-seq experiment presented in Figure 2D.

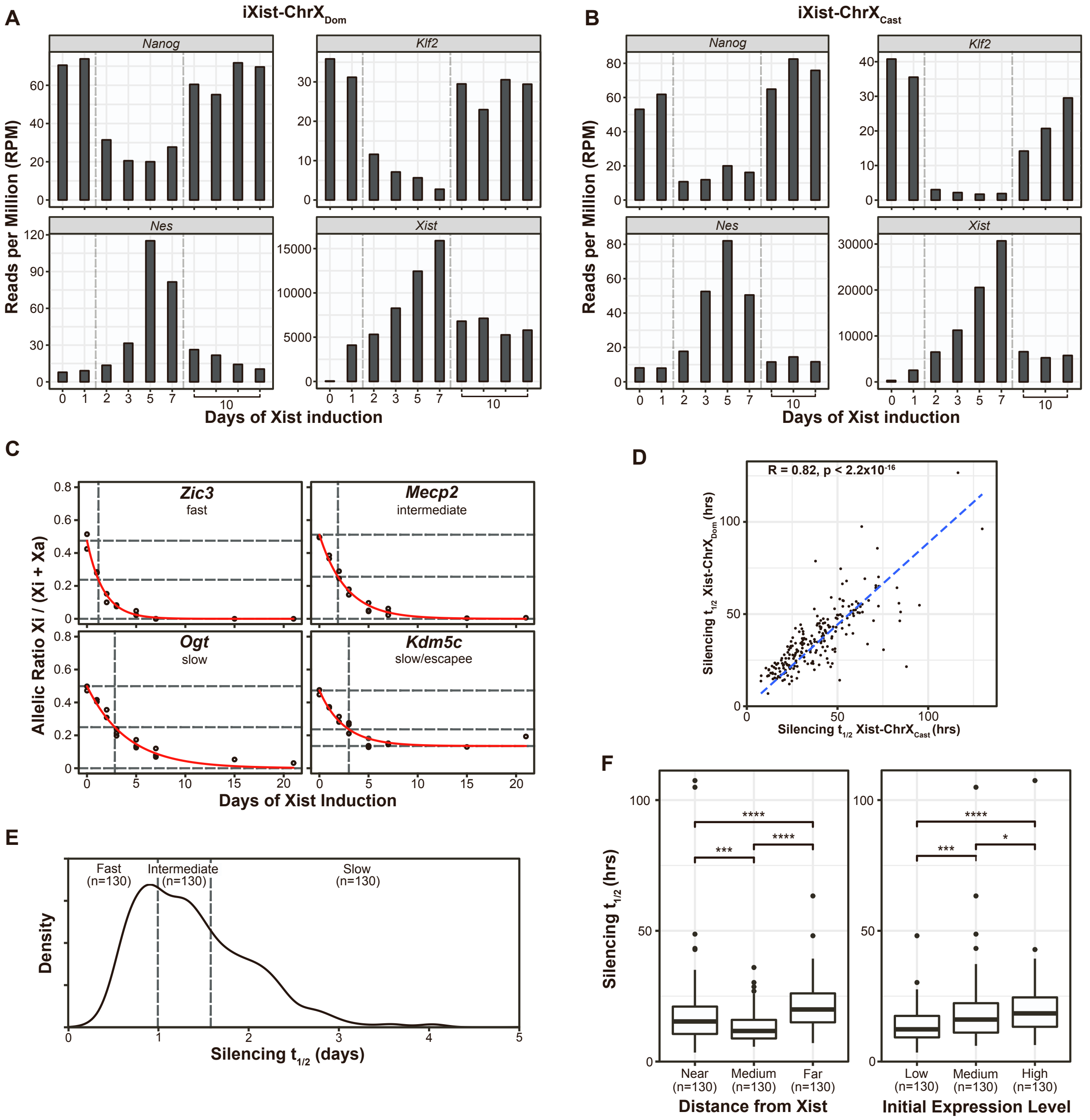

Figure S5 Modelling of silencing kinetics over a timecourse of Xist induction with NPC differentiation. Related to Figure 4.

(A,B) Relative levels of selected marker gene expression and chromatin-associated Xist for the (A) iXist-ChrX$_{Dom}$ or (B) iXist-ChrX$_{Cast}$ NPC timecourse and undifferentiated day 10 Dox-induced mESC samples shown in with Figure 4A,B.

(C) Silencing trajectories for individual example genes in the iXist-ChrX$_{Cast}$ cell line. *Zic3*, *Mecp2*, *Ogt* are fast, intermediate, and slow silencing respectively. *Kdm5c* is an escapee but is also classified as slow silencing by its initial silencing kinetics. Horizontal lines represent parameters of y_0 (initial allelic ratio), 1/2 y_0, and y_f (final allelic ratio) respectively, and the vertical line is placed at the calculated $t_{1/2}$ (halftime) for each gene.

(D) Scatter plot comparing calculated silencing halftimes for each gene amenable to allelic analysis of silencing kinetics in both iXist-ChrX$_{Dom}$ and iXist-ChrX$_{Cast}$ lines (n=235). Halftimes are strongly

correlated with a Spearman's rank correlation coefficient of R = 0.82.

(E) Density plot of gene silencing halftimes in iXist-ChrX$_{Cast}$, allowing for classification of genes into equal-sized groups of fast, intermediate, and slow silencing genes.

(F) Boxplots comparing silencing halftimes between subsets of genes based on distance from the *Xist* locus (left) or initial expression level in mESCs (right) (see STAR Methods). Significance of individual comparisons is determined by unpaired two-sample Wilcoxon rank-sum test. *, **, ***, **** indicate p below 0.05, 0.01, 0.001 and 0.0001 respectively.

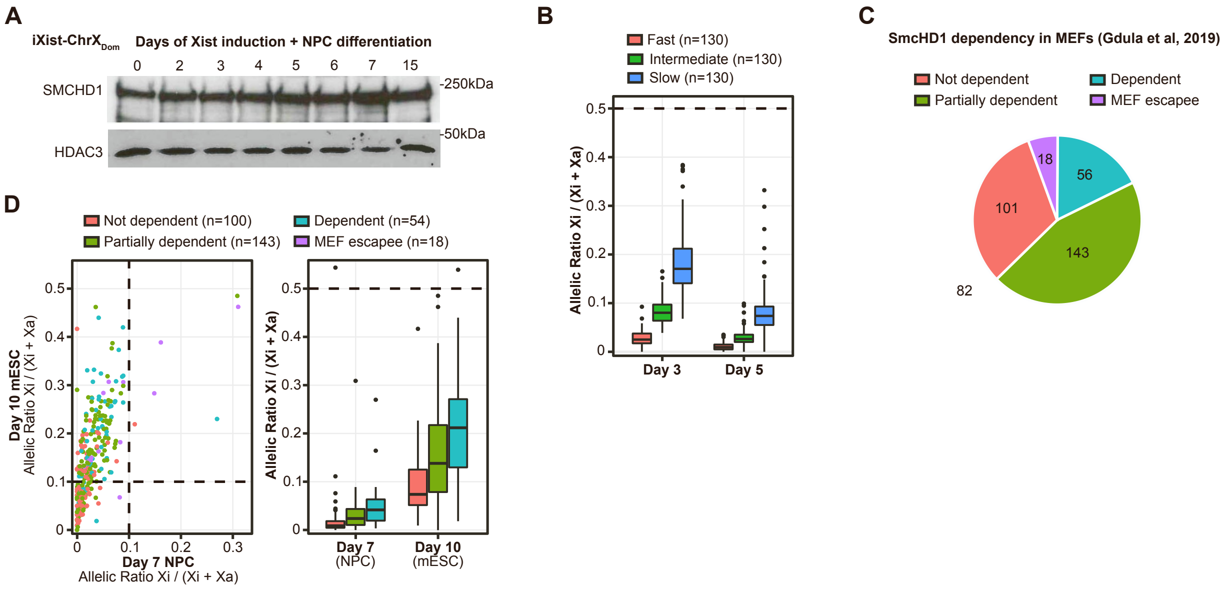

Figure S6 Slow genes are incompletely silenced at the time window when SmcHD1 is recruited to Xi. Related to Figure 5.

A) Western blot analysis of SmcHD1 protein levels in iXist-ChrX$_{Dom}$ cells during the differentiation timecourse. Note that the amount of SmcHD1 protein present in mESCs (day 0) is comparable to the amount of SmcHD1 detected during differentiation. HDAC3 is included as a loading control. Molecular weight markers are shown on the right.

B) Boxplots summarizing allelic ChrRNA-seq data from iXist-ChrX$_{Cast}$ NPC day 3 and day 5. Boxes compare kinetic classes of genes to demonstrate incomplete silencing of intermediate and slow genes during the time window of SmcHD1 recruitment to Xi.

C) Pie chart depicting the number of genes in each class of SmcHD1 dependency, as defined from ChrRNA-seq analysis of XX mouse embryo fibroblast (MEF) lines derived from SmcHD1 null embryos. 82 genes amenable to analysis in the iXist-ChrX$_{Cast}$ cellular model were not previously categorized due to insufficient expression in MEFs.

D) Scatter plot comparing individual gene allelic ratios between iXist-ChrX$_{Cast}$ undifferentiated mESC day 10 and NPC day 7. Genes are coloured according to SmcHD1 dependency categories. Dashed lines indicate trace allelic ratios of 0.1. Adjacent boxplots compare iXist-ChrX$_{Cast}$ undifferentiated mESC day 10 and NPC day 7 by SmcHD1 dependency categories. MEF escapees are excluded due to insufficient number of genes.

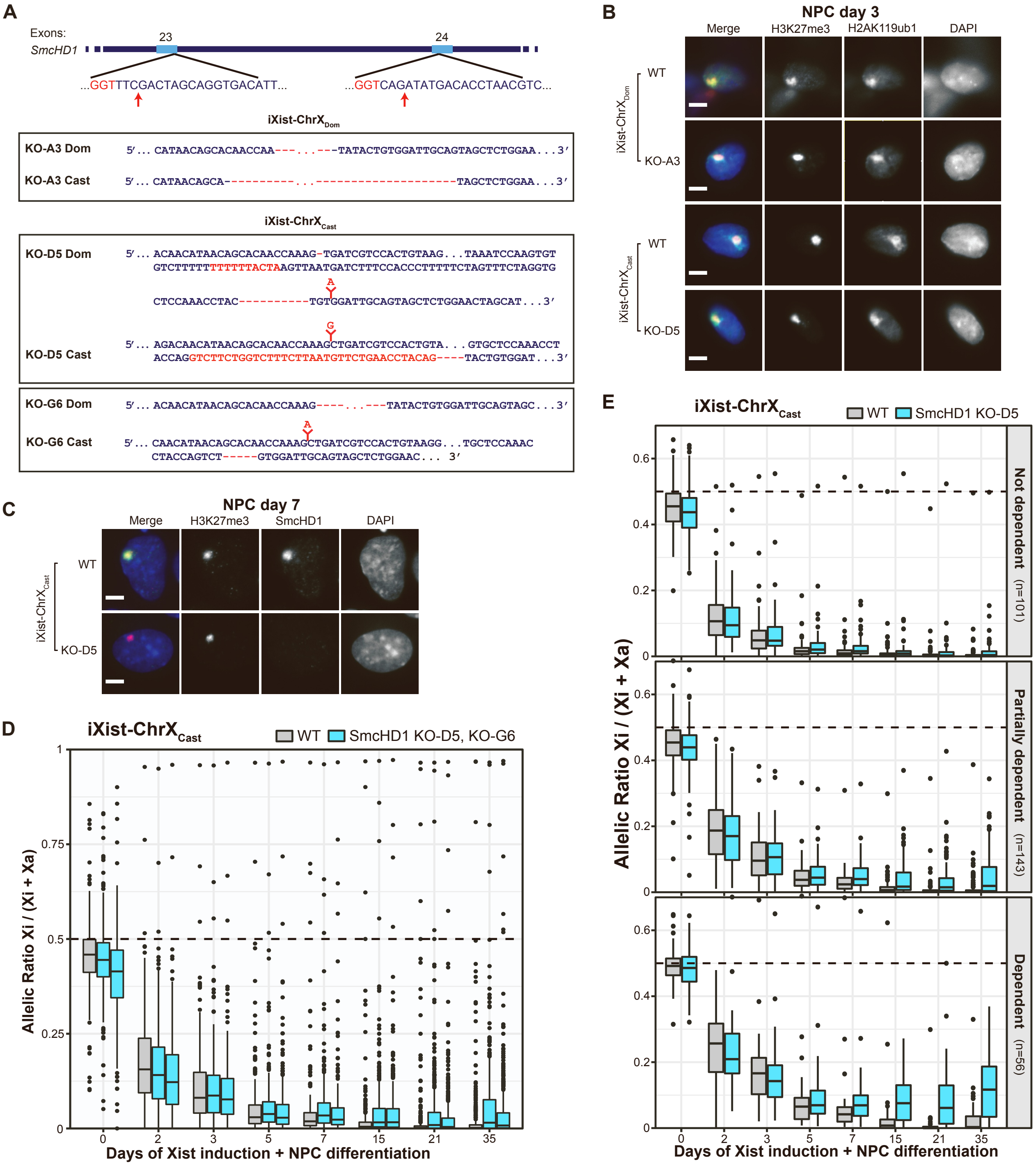

Figure S7 Characterization of SmcHD1 KO cell lines. Related to Figure 6.

A) Strategy for CRISPR-Cas9-mediated mutagenesis of the *SmcHD1* gene. The positions of guide RNAs relative to *SmcHD1* exons are shown, with PAM sequences (red font) and predicted cleavage sites (red arrows) indicated. Two sgRNAs for exons 23 and 24 were used to generate mutant clones in iXist-ChrX$_{Dom}$ and iXist-ChrX$_{Cast}$. Mutated sequences of *Domesticus* (Dom) and *Castaneous* (Cast) alleles are shown for each KO line. Two independent KO lines were created and analysed for iXist-ChrX$_{Cast}$. Deletions are shown by red dashes and insertions/mutations are shown in red font.

B) Immunofluorescence analysis of Polycomb modifications H3K27me3 and H2AK119ub1 in WT

and SmcHD1 KO clones in NPC day 3, confirming Xi domain formation in KO cells. Scale bar is 5μm.

C) Immunofluorescence analysis of SmcHD1 in WT and iXist-ChrX$_{Cast}$ KO clone D5 at NPC differentiation day 7, demonstrating the absence of SmcHD1 over the Xi (as marked by an enriched domain of the Polycomb modification H3K27me3). H3K27me3 is shown in magenta, SmcHD1 in cyan, and DNA in blue (DAPI) in the merged image on the left. Scale bar is 5 μm.

D) Boxplots summarizing allelic ChrRNA-seq analysis of X-linked gene expression in iXist-ChrX$_{Cast}$ WT and two independent SmcHD1 KO clones (D5 and G6). Boxes of days 3, 5 and 7 of Xist induction are averaged from three replicate timecourse experiments, day 2 from duplicates, and days 0 and 15 - 35 of SmcHD1 KO from individual samples.

E) Boxplots summarizing allelic ChrRNA-seq analysis of X-linked gene expression in iXist-ChrX$_{Cast}$ WT and SmcHD1 KO (clone D5), with subsets of genes separated by SmcHD1 dependence categories. Boxes of days 3, 5 and 7 of Xist induction are averaged from three replicate timecourse experiments, day 2 from duplicates, and days 0 and 15 - 35 of SmcHD1 KO from individual samples.

Name	Sequence	Source	Purpose
SPOC_sgRNA_F_GW135	caccgCCCCACTGCGGATCGCCCAG	Rodermund et al, 2021	sgRNA to *Spen* SPOC domain sequence
Pcgf3_Nter_sgRNA_F_JB021	caccGAATGAGGTAGCCGCTGCAC	This study	sgRNA to N-ter *Pcgf3*
Pcgf5_Nter_sgRNA_F_JB033	caccGACCTCGAATGGCTACCCAA	This study	sgRNA to N-ter *Pcgf5*
SmcHD1_sgRNA_1412	caccgTTACAGTGGACGATCAGCTT	Gdula et al, 2019	sgRNA to SmcHD1 exon 23
SmcHD1_sgRNA_1414	caccgCTGCAATCCACAGTATAGAC	Gdula et al, 2019	sgRNA to SmcHD1 exon 24
SPOCmut_Screen12_R_GW142	CAGCAGCAGGCAGTAGTCGG	Rodermund et al, 2021	$SPEN^{SPOCmut}$ genotyping/screening
SPOCmut_Screen1_F_GW143	GGATCGCCCAGGCCATGGCA	Rodermund et al, 2021	$SPEN^{SPOCmut}$ genotyping/screening
SPOCmut_Screen2_F_GW141	GGGACACCACAACGGCCTGTG	Rodermund et al, 2021	$SPEN^{SPOCmut}$ genotyping/screening
SmcHD1_TNK209	GTATTTAATTACATAATTTCTCAATAGGTCCC	This study	genotyping/screening KO
SmcHD1_TNK228	AAATCTGAGTATTAATAACTGTCAC	This study	genotyping/screening KO
SmcHD1_TNK290	GGCAGCAGTGTTAGCTGTGG	Gdula et al, 2019	genotyping/screening KO
SmcHD1_TNK291	AATGAAAGCAGTAGCTTACAGG	Gdula et al, 2019	genotyping/screening KO
SmcHD1_TNK418_Dom	TGTTTTTAAGAGAACCATAAATAC	This study	characterisation KO, *Domesticus*-specific primer
GeneDesert_qPCR_F_TZ246	TGCATGAGCAGAGGCCTAGG	This study	Polycomb ChIP qPCR verification
GeneDesert_qPCR_R_TZ247	AGAAGTGCAAGCTCAGAACCTT	This study	Polycomb ChIP qPCR verification
Cdx2_Prom_F_TZ58	ACCACCTTCTGCCTGAGAATGTAC	This study	Polycomb ChIP qPCR verification
Cdx2_Prom_R_TZ59	CCTCCAATCACAGGTTCAAAGACT	This study	Polycomb ChIP qPCR verification

Table S1 Oligonucleotides used in this study. Related to STAR Methods.
